# Amino Acid Profiles in Older Adults with Frailty: Secondary Analysis from MetaboFrail and BIOSPHERE Studies

**DOI:** 10.3390/metabo13040542

**Published:** 2023-04-10

**Authors:** Riccardo Calvani, Anna Picca, Leocadio Rodriguez-Mañas, Matteo Tosato, Hélio José Coelho-Júnior, Alessandra Biancolillo, Olga Laosa, Jacopo Gervasoni, Aniello Primiano, Lavinia Santucci, Ottavia Giampaoli, Isabelle Bourdel-Marchasson, Sophie C. Regueme, Alan J. Sinclair, Andrea Urbani, Francesco Landi, Giovanni Gambassi, Federico Marini, Emanuele Marzetti

**Affiliations:** 1 Fondazione Policlinico Universitario “Agostino Gemelli” IRCCS, 00168 Rome, Italy; 2 Department of Geriatrics and Orthopedics, Università Cattolica del Sacro Cuore, 00168 Rome, Italy; 3 Department of Medicine and Surgery, LUM University, 70010 Casamassima, Italy; 4 Servicio de Geriatría, Hospital Universitario de Getafe, 28905 Getafe, Spain; 5 Centro de Investigación Biomédica en Red “Fragilidad y Envejecimiento Saludable” (CIBERFES), Instituto de Salud Carlos III, 28029 Madrid, Spain; 6 Department of Physical and Chemical Sciences, Università degli Studi dell’Aquila, 67100 L’Aquila, Italy; 7 Geriatric Research Group, Biomedical Research Foundation at Getafe University Hospital, 28905 Getafe, Spain; 8 Department of Chemistry, Sapienza Università di Roma, 00185 Rome, Italy; 9 Clinical Gerontology Department, Bordeaux University Hospital, 33000 Bordeaux, France; 10 CRMSB, CNRS UMR 5536, Université de Bordeaux, 33000 Bordeaux, France; 11 CHU Bordeaux, Pole Gérontologie Clinique, 33000 Bordeaux, France; 12 Foundation for Diabetes Research in Older People (fDROP), King’s College, London WC2R 2LS, UK

**Keywords:** aging, geroscience, metabolic profiling, metabolism, metabolomics, muscle wasting, protein, physical function, sarcopenia, systems biology

## Abstract

An altered amino acid metabolism has been described in frail older adults which may contribute to muscle loss and functional decline associated with frailty. In the present investigation, we compared circulating amino acid profiles of older adults with physical frailty and sarcopenia (PF&S, *n* = 94), frail/pre-frail older adults with type 2 diabetes mellitus (F-T2DM, *n* = 66), and robust non-diabetic controls (*n* = 40). Partial least squares discriminant analysis (PLS–DA) models were built to define the amino acid signatures associated with the different frailty phenotypes. PLS–DA allowed correct classification of participants with 78.2 ± 1.9% accuracy. Older adults with F-T2DM showed an amino acid profile characterized by higher levels of 3-methylhistidine, alanine, arginine, ethanolamine, and glutamic acid. PF&S and control participants were discriminated based on serum concentrations of aminoadipic acid, aspartate, citrulline, cystine, taurine, and tryptophan. These findings suggest that different types of frailty may be characterized by distinct metabolic perturbations. Amino acid profiling may therefore serve as a valuable tool for frailty biomarker discovery.

## 1. Introduction

Frailty is a multifaceted condition characterized by a decreased homeostatic reserve and a reduced resistance to stressors, which lead to an increased risk of negative health outcomes [1]. Frailty has many phenotypic manifestations reflecting its heterogeneous pathophysiology, which encompasses multilevel alterations ranging from subcellular processes to socioeconomic determinants [2]. Frailty, especially in its physical domain, shares common risk factors and clinical manifestations with major age-related conditions, including sarcopenia and type 2 diabetes mellitus (T2DM) [3,4,5]. Muscle wasting may indeed represent the common ground upon which frailty and associated conditions develop and progress [6,7,8]. The recognition of muscle failure as the biological substratum of frailty and associated diseases may help identify mechanisms and biomarkers associated with these conditions, and develop new therapeutics [9,10,11].

Amino acid metabolism plays a central role in both energy homeostasis and muscle trophism, and modulates critical processes such as inflammation, insulin sensitivity, redox balance, and stem cell function [12,13,14,15,16]. Alterations in these processes, in turn, are associated with the development of frailty and degenerative diseases. Targeted metabolomics approaches have been used to measure circulating amino acid levels and identify amino acid profiles associated with muscle wasting disorders, T2DM, and frailty [17,18,19,20].

Recently, within the “BIOmarkers associated with Sarcopenia and PHysical frailty in EldeRly pErsons” (BIOSPHERE) and “Metabolic biomarkers of frailty in older people with type 2 diabetes mellitus” (MetaboFrail) studies, we showed that specific amino acid profiles identified older adults with physical frailty and sarcopenia (PF&S) [21] and pre-frail/frail older adults with T2DM (F-T2DM) [22], respectively. In the present study, we conducted secondary analyses to characterize similarities and differences in the amino acid profiles of older adults with PF&S and F-T2DM, and obtain further insights into the relationship between protein/amino acid dyshomeostasis and frailty.

## 2. Materials and Methods

### 2.1. Study Participants

The present investigation included participants enrolled in MetaboFrail and BIOSPHERE studies [23,24]. MetaboFrail was designed within the “Multi-modal Intervention in Diabetes in Frailty” (MID-Frail) project [23,25,26]. The MID-Frail Consortium included clinical and research centers across seven European countries (Belgium, Czech Republic, France, Germany, Italy, Spain, and United Kingdom). The aim of the main project was to optimize the medical management of older adults with F-T2DM through the adoption of a multicomponent intervention (strength training plus personalized nutritional counseling). A multicenter randomized clinical trial was conducted to test the efficacy of the proposed intervention at increasing the short physical performance battery (SPPB) score compared with standard of care in older adults with F-T2DM (ClinicalTrials.gov identifier: NCT01654341) [25,26]. For the MetaboFrail substudy, a cohort of MID-Frail participants from Spain and France was recruited [23,25,26]. Participants of MetaboFrail were men and women aged 70+ years, diagnosed with T2DM for more than two years, and who were pre-frail or frail according to the criteria proposed by Fried et al. [27]. BIOSPHERE was conceived as an observational study to identify biomarkers for PF&S through a multi-marker strategy [24]. BIOSPHERE was conducted at the Department of Geriatrics and Orthopedics of the Università Cattolica del Sacro Cuore, Rome, Italy (IRB no. 8498/15). The study protocol is detailed elsewhere [24]. BIOSPHERE participants were older adults aged 70+ with PF&S. PF&S was operationalized as the co-occurrence of reduced physical performance, defined as an SPPB score from 3 to 9 [28], and low appendicular lean mass according to the criteria established by the Foundation for the National Institutes of Health sarcopenia project [29]. PF&S was further characterized by a retained ability to walk 400 m in 15 min at a usual pace (i.e., absence of mobility disability) [30].

Control participants were enrolled at the geriatric outpatient clinic of the Fondazione Policlinico A. Gemelli IRCCS at the Università Cattolica del Sacro Cuore (Rome, Italy) and had the following characteristics: (a) 70+ years; (b) no diagnosis of T2DM; (c) a summary score on the SPPB > 9; and (d) no mobility disability. All participants provided written informed consent prior to enrollment. The study was conducted in accordance with the recommendations by the International Council for Harmonization of Technical Requirements for Pharmaceuticals for Human Use Good Clinical Practice and the principles of the Declaration of Helsinki.

### 2.2. Determination of Serum Concentrations of Amino Acids and Derivatives

Samples for serum determinations were collected by blood drawing after overnight fasting and processed following standard procedures for serum separation and storage. Serum levels of 37 analytes, including amino acids and intermediates, were determined by ultraperformance liquid chromatography/mass spectrometry (UPLC/MS). Methods for the UPLC/MS analysis have been thoroughly detailed in previous publications [21,22]. Briefly, 50 μL of serum were added to 100 μL 10% *w*/*v* sulfosalicylic acid containing a mixture of internal standards (50 μM; Cambridge Isotope Laboratories, Inc., Tewksbury, MA, USA). The solution was subsequently centrifuged at 1000× *g* for 15 min. The supernatant (10 μL) was mixed with borate buffer (70 μL) and AccQ Tag reagents (20 μL) (Waters Corporation, Milford, MA, USA). The solution was heated at 55 °C for 10 min. The chromatographic separation was performed using CORTECS UPLC C18 column 1.6 μm 2.1 × 150 mm (Waters Corporation). The elution flow rate was set at 500 μL/min with a linear gradient (9 min) from 99:1 to 1:99 water 0.1% formic acid/acetonitrile 0.1% formic acid. Detection was carried out by single quadrupole mass spectrometer (ACQUITY QDa, Waters Corporation) using positive electrospray ionization mode. Amino acid controls (level 1 and level 2), manufactured by the MCA laboratory of the Queen Beatrix Hospital (The Netherlands), were used to monitor the analytical process. Analyte concentrations were determined by comparison with values obtained from individual standard curves. Standard curve values were 0.5–2.5–125–250–500 μmol/L for all amino acids, except for cystine for which the following values were used: 1–5–50–250–500–1000 μmol/L. Data analysis was performed using the TargetLynx software (Waters Corporation).

### 2.3. Statistical Analysis

Normal distribution of data was assessed via the Shapiro–Wilk test. Personal, anthropometric, and functional characteristics of participants are summarized as mean ± standard deviation for continuous variables and absolute values (percentages) for categorical variables. Comparisons among PF&S, F-T2DM, and controls were performed by one-way analysis of variance with post hoc tests when appropriate and χ^2^ statistics for continuous and categorical variables, respectively. Analyses were performed using Jamovi freeware version 2.0.0.0 (The Jamovi project, 2021; retrieved from https://www.jamovi.org; accessed on 20 February 2023). Multivariate classification models, based on partial least squares discriminant analysis (PLS–DA) [31,32] and soft independent modeling of class analogies (SIMCA), were built to define similarities and differences in circulating amino acid patterns among F-T2DM, PF&S, and control participants. 

#### 2.3.1. Partial Least Squares Discriminant Analysis

PLS−DA is a classification technique that was introduced to build discriminant models also in cases where the matrix of predictors is ill-conditioned (e.g., having more variables than samples or variables being highly correlated). This is enabled by the exploitation of the advantages of the PLS algorithm through the transformation of a classification problem into a regression by suitably coding the target response vector. The PLS algorithm overcomes the limitations of ill-conditioning by projecting the predictor matrix X onto a low-dimensional subspace of orthogonal latent variables. The projection is accomplished through a suitable matrix of weights R identifying the directions of maximum covariance between the predictors and the response y. The result is a matrix of scores T (coordinates of the samples onto the latent variables subspace):

T = XR(1)

The response y to be predicted is then expressed as a function of the scores T, according to:

y = Tq (2)

q being the regression coefficients.

The same approach can be used for classification of two or more groups by using a dummy binary y coding for class belonging. When the problem involves only two classes, y is a vector whose elements can be either 1 (class 1) or 0 (class 2). In the case of more than two categories, the responses are collected in a binary matrix Y having as many columns as the number of classes. In the latter case, each row of Y contains all zeros except for the column corresponding to the category of the sample, where a value of 1 is present. In both cases, linear discriminant analysis is applied to the predicted values of the response to achieve the final classification.

In the present study, classification models were first built to evaluate differences in amino acid profiles between PF&S (y = 1) and F-T2DM (y = 0). Then, PLS–DA models were built to explore differences between the three groups, and the corresponding rows of the dummy Y matrix were coded as follows: [1 0 0] for controls, [0 1 0] for PF&S, and [0 0 1] for F-T2DM. 

Model validation was achieved through repeated double cross-validation (DCV) [33]. In DCV, all available samples are arranged into two cross-validation loops nested into one another. Model selection (i.e., choosing the optimal number of latent variables) is based on the classification error estimated on the inner loop. The evaluation of model performances in independent validation samples is carried out on the outer loop, which mimics an external test set. The procedure is repeated a suitable number of times (50, in the present study), changing at each iteration the distribution of samples in the different cancelation groups. This allows calculating confidence intervals for all model parameters and figures of merit. Analyses were performed using in-house routines running under MATLAB R2015b environment (The MathWorks, Natick, MA, USA) and freely downloadable at https://www.chem.uniroma1.it/romechemometrics/research/algorithms/plsda (accessed on 20 February 2023).

#### 2.3.2. Soft Independent Modeling of Class Analogies

SIMCA is a chemometric class modeling technique and, as such, it focuses on one category at a time, trying to capture its salient features by means of an individual model. Classification translates into checking how likely it is for an individual to be part of that specific category, usually by computing some sort of distance to the model [34,35]. Mathematically, SIMCA builds a model for each class of participants (in our case, PF&S, F-T2DM, and controls) using principal component analysis (PCA) only on the data of the category of interest. The decision of whether an individual should be considered as belonging to that class or not (i.e., be accepted by the class model or not) relies on calculating a distance to the model according to the formula:

(3) $$d_{ic}=\sqrt{{{(T}_{ic,red}^{2})}^{2}+{\left(Q_{ic,red}\right)}^{2}}$$

where $T_{ic,red}^{2}$ is the Mahalanobis distance of the *i*th sample from the center of the PCA space calculated for class *c*, $Q_{ic,red}$ is the orthogonal distance (residual) of the sample to its projection on the PCA space of class *c*, and the subscript red indicates that the two statistics are normalized by their respective 95th percentile. Acceptance or rejection of the unknown samples is based on imposing a threshold to the distance described in Equation (3), which is usually equal to $\sqrt{2}$. Accordingly, if $d_{ic}<\sqrt{2}$, the individual is accepted by the class model, otherwise it is rejected. Sensitivity and specificity of the model are then calculated. Analyses were performed using in-house routines running under MATLAB R2015b environment (The MathWorks). 

## 3. Results

### 3.1. Characteristics of Study Participants

The present study included data from 94 older adults with PF&S, 66 with F-T2DM, and 40 controls. The main characteristics of participants according to frailty categories are listed in Table 1. Participants with PF&S and F-T2DM did not differ for age or body mass index (BMI) values, while controls were significantly younger and had lower BMI. Participants with PF&S were mostly women, while F-T2DM and controls had a similar sex distribution. As expected, SPPB scores were significantly lower in PF&S (mean difference = −4.2) or F-T2DM (mean difference = −2.8) than controls. In addition, the mean SPPB score was lower in PF&S than F-T2DM participants (mean difference = −1.4).

### 3.2. Participant Classification by Partial Least Squares Discriminant Analysis

**Figure 1:**
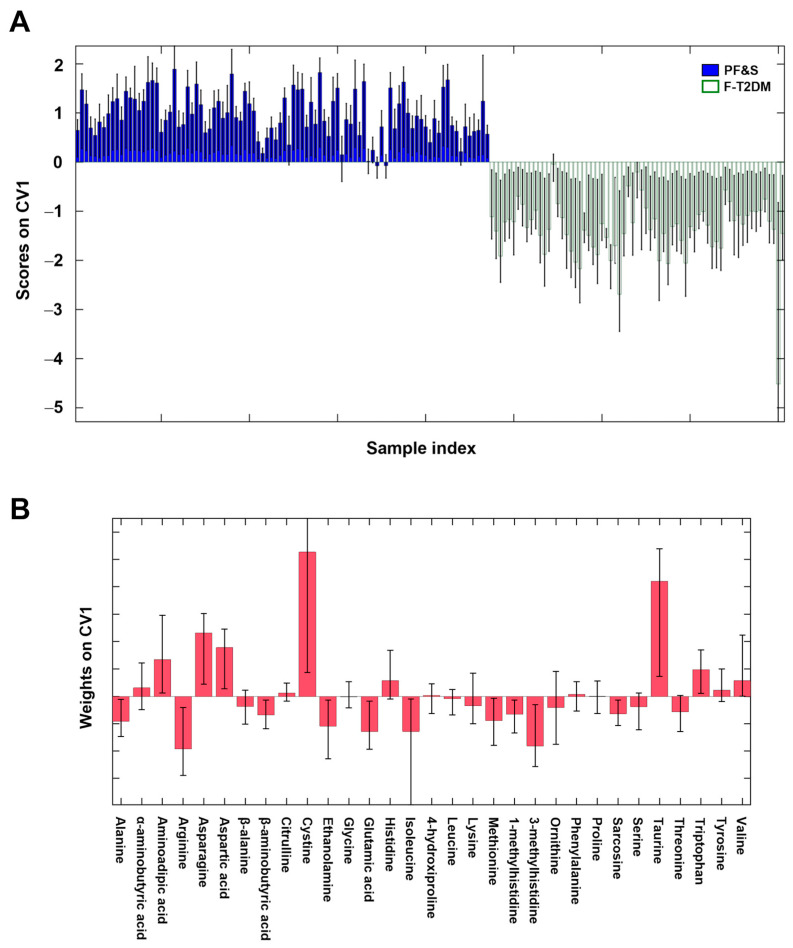
Participant classification according to serum amino acid profiles based on partial least squares discriminant analysis. Outer loop sample scores (**A**) and variable weights (**B**) along the only canonical variate of model. Abbreviations: CV1, canonical variate 1; F-T2DM, pre-frailty/frailty with type 2 diabetes mellitus; PF&S, physical frailty and sarcopenia.

The data matrix used to build PLS–DA models included 31 out of 37 amino acids (Figure S1) because six analytes had concentrations below the lower limit of quantitation (i.e., anserine, carnosine, cystathionine, γ-aminobutyric acid, phosphoethanolamine, and phosphoserine). The first PLS–DA model was built to identify similarities and differences in circulating amino acid patterns between participants with PF&S and F-T2DM (Figure 1). The optimal PLS–DA model complexity included 5 ± 2 latent variables. The model allowed the prediction of participant class belonging with 98.8 ± 0.4% accuracy, corresponding to 99.6 ± 0.5% and 97.7 ± 0.9% correct classification rates for PF&S and F-T2DM, respectively. The non-parametric estimation of the distribution of these figures of merit under the null hypothesis by permutation testing indicated that they were statistically significant (*p* < 0.001).

The remarkable difference between the amino acid profiles of participants with PF&S and F-T2DM, and the contribution of individual analytes to the discrimination can be appreciated by inspecting the sample scores along the only canonical variate (CV) (i.e., direction of maximum discrimination) of the model and the corresponding variable weights defining the projection (Figure 1).

The variables that mostly contributed to participant classification were alanine, arginine, β-aminobutyric acid, ethanolamine, glutamic acid, isoleucine, methionine, 1- and 3-methylhistidine, and sarcosine (higher in F-T2DM), and aminoadipic acid, asparagine, aspartic acid, cystine, taurine, and tryptophan (higher in PF&S).

A PLS–DA model was then built to compare the three classes of participants (i.e., PF&S, F-T2DM, and controls). The optimal model complexity was found to be 10 ± 5 latent variables, yielding an average classification accuracy of 78.2 ± 1.9%. The correct classification rates were 72.9 ± 2.8% for PF&S, 95.5 ± 2.1% for F-T2DM, and 61.4 ± 4.6% for controls. DCV indicated that the results were statistically significant (*p* < 0.001).

**Figure 2:**
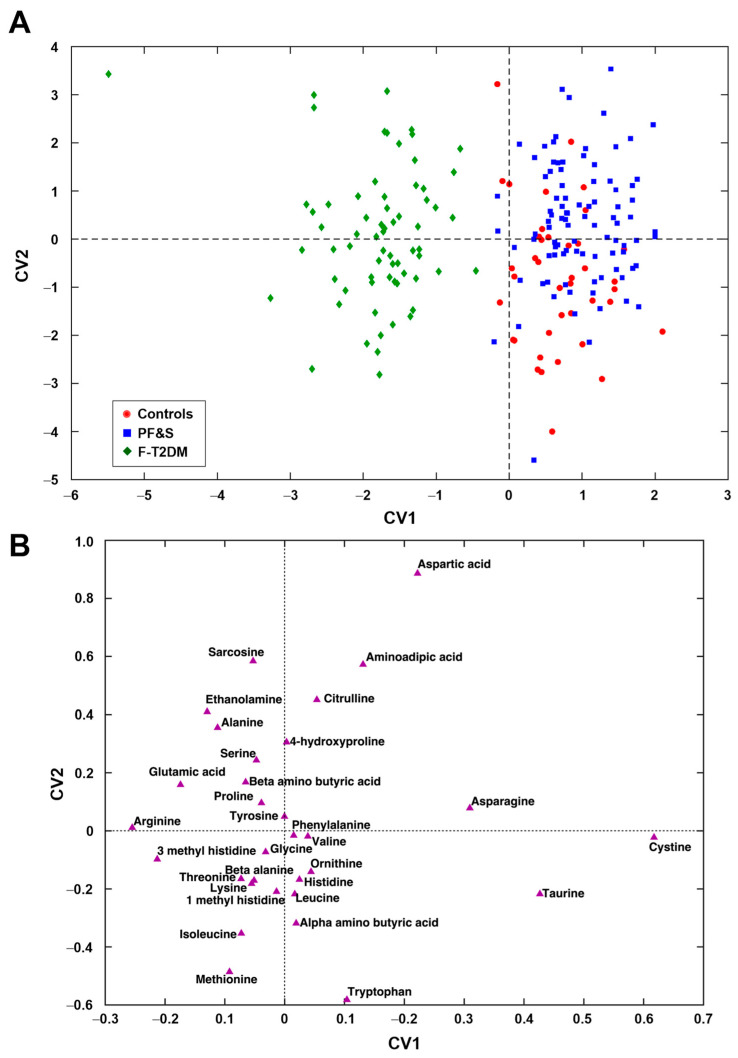
Participant classification according to serum amino acid profiles based on partial least squares discriminant analysis. Outer loop sample scores (**A**) and variable weights plot (**B**) along the two canonical variates of model. Abbreviations: CV, canonical variate; F-T2DM, pre-frailty/frailty with type 2 diabetes mellitus; PF&S, physical frailty and sarcopenia.

Figure 2A, which depicts the projection of participants onto the space spanned by the only two CVs of the PLS–DA model, shows a clear separation of participants with F-T2DM from those with PF&S and controls along CV1. A differentiation between participants with PF&S and controls can be observed along CV2, although the separation is not as evident.

The examination of the weights plot (Figure 2B) allows identifying the variables that mostly contributed to the differentiation among the three classes of participants and their relationships. To simplify, variables lying farthest from the origin are those contributing the most to the definition of the CVs and, therefore, to sample discrimination. Variables lying close to one another are positively correlated, while those lying on the opposite side of the plot with respect to the origin are negatively correlated. The simultaneous inspection of scores (Figure 2A) and weights plots (Figure 2A) makes it possible to associate discrimination between participant classes with differences in concentrations of specific analytes, which are therefore the most distinctive. Participants that occupy a position in the scores plot where one or a group of variables lies in the corresponding weights plot have the highest value(s) of this(ese) variable(s). Accordingly, participants with F-T2DM showed higher levels of 3-methylhistidine, alanine, arginine, ethanolamine, and glutamic acid. PF&S and control participants were discriminated based on serum concentrations of aminoadipic acid, aspartate, cystine, taurine, and tryptophan (Figure S1).

### 3.3. Participant Classification According to Soft Independent Modeling of Class Analogies Analysis

**Figure 3:**
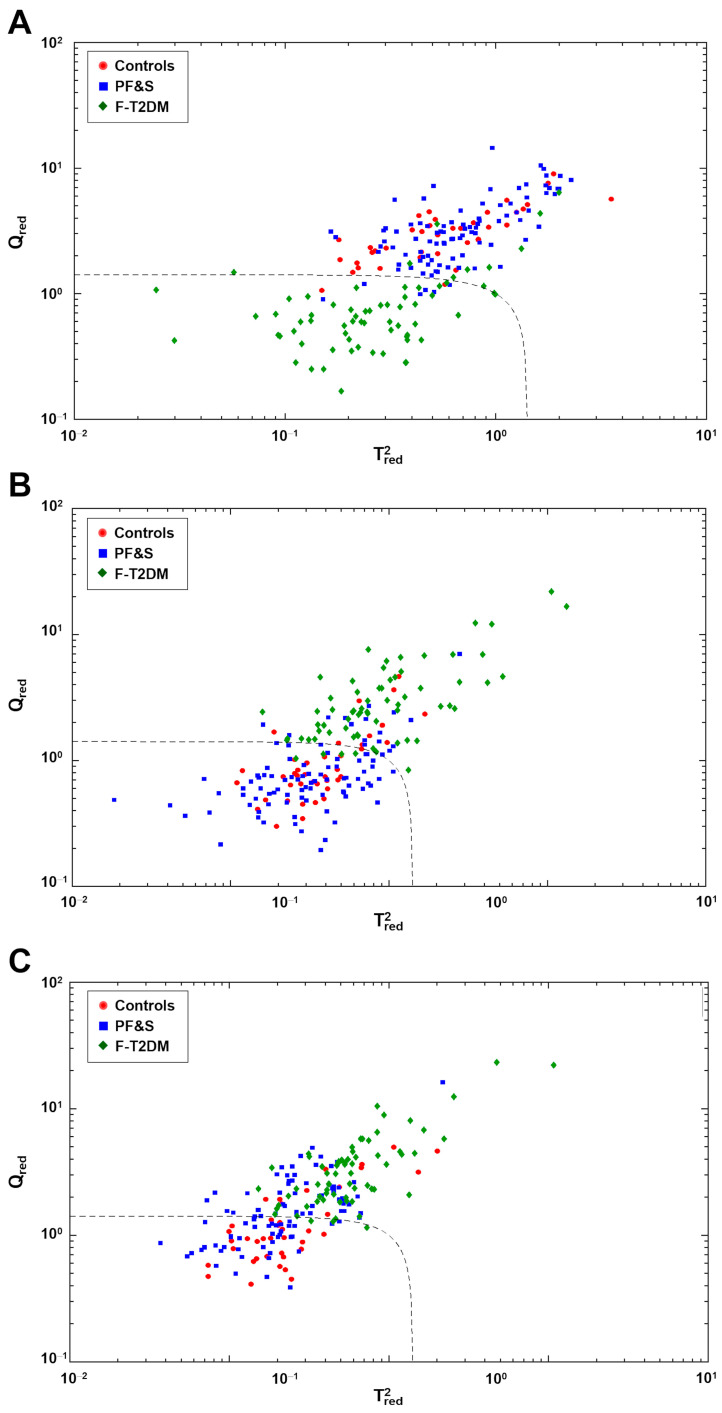
Participant classification according to serum amino acid profiles based on soft independent modeling of class analogies analysis. Projection of the outer loop sample onto the space identified by the values of $T_{red}^{2}$ and $Q_{red}$ for the class models: (**A**) pre-frailty/frailty with type 2 diabetes mellitus (F-T2DM); (**B**) physical frailty and sarcopenia (PF&S); and (**C**) controls.

The existence of specific amino acid profiles associated with frailty categories was further tested by SIMCA class modeling. Separate SIMCA models were built and validated by DCV for one participant group at a time. Sensitivity was determined based on the model ability to correctly recognize participants as belonging to their actual category. Specificity was calculated as the percentage of participants correctly rejected. The optimal complexity of the PCA model for each class was found to be 8 ± 1, 10 ± 1, and 7 ± 1 components, respectively. The results of SIMCA analysis on the outer loop samples of DCV are depicted in Figure 3. The dashed black line corresponds to the decision threshold $d_{ic}<\sqrt{2.}$ The SIMCA model built for F-T2DM had 85.1% sensitivity and 94.0% overall specificity, corresponding to 93.6% specificity versus PF&S and 95.0% versus controls (Figure 3A). The model built for PF&S had 81.9% sensitivity and 68.2% total specificity (94.0% versus F-T2DM and 25.0% versus controls) (Figure 3B). Finally, the model built for controls showed 70.0% sensitivity and 71.4% overall specificity (95.5% versus F-T2DM and 54.3% versus PF&S) (Figure 3C). Collectively, the results of SIMCA analysis confirmed the existence of a clear difference between the amino acid profiles of participants with F-T2DM compared with PF&S and controls. A less obvious difference was found between PF&S and controls.

## 4. Discussion

In the present investigation, we showed that specific amino acid profiles are associated with different types of frailty in community-dwelling older adults. Participants with F-T2DM had a serum amino acid signature that was markedly different from those with PF&S and controls. Older adults with PF&S could be discriminated from controls based on their circulating amino acid profile, albeit with less accuracy. The amino acid profile associated with F-T2DM was characterized by higher levels of 3-methylhistidine, alanine, arginine, ethanolamine, and glutamic acid. PF&S and control participants were discriminated based on serum concentrations of aminoadipic acid, asparagine, aspartic acid, cystine, taurine, and tryptophan.

3-methylhistidine derives from the post-translational methylation of histidine residues of actin and myosin [36,37]. Following muscle protein breakdown, 3-methylhistidine is released into the circulation and excreted in urine [38]. Hence, 3-methylhistidine has been proposed as a marker of myofibrillar proteolysis in muscle wasting disorders and T2DM [39,40,41,42,43]. High circulating levels of 3-methylhistidine have been found in frail older inpatients and community-dwellers [20,44].

Alanine and glutamic acid are two non-essential gluconeogenic amino acids involved in an interorgan metabolic network that regulates insulin sensitivity and energy homeostasis in insulin-sensitive tissues, such as skeletal muscle and liver [45,46,47]. Alterations in circulating alanine and glutamic acid levels have been described in chronic disease (e.g., T2DM, chronic obstructive pulmonary disease) and experimental models of muscle atrophy [48,49,50]. Elevated concentrations of glutamic acid may also promote oxidative stress and contribute to glucose toxicity in pancreatic β-cells [51]. In a systematic review and meta-analysis of prospective cohort studies involving 71,196 participants across US, Europe, and Asia, higher circulating levels of alanine and glutamic acid were associated with a greater risk of T2DM [18]. In the Baltimore Longitudinal Study of Aging, higher plasma concentrations of alanine and glutamic acid were linked to increased odds of abnormal fasting glucose [52]. Moreover, serum levels of glutamic acid were significantly higher in frail older adults compared with non-frail peers and young adults in a cross-sectional study involving 166 community-dwellers aged 20–93 years, living in the Baltimore area [53].

Ethanolamine is involved in the cytidine 5′-diphosphate (CDP)–ethanolamine pathway, one of the main mechanisms through which glycerophospholipids and biological membranes are synthetized in mammalian cells [54,55]. Perturbations in the CDP–ethanolamine pathway or its intermediates in skeletal muscle have been associated with tissue damage, mitochondrial dysfunction, and altered glucose homeostasis [56,57]. 

Arginine metabolism is involved in the regulation of key biological processes, including immune and vascular health, neurotransmission, and respiratory function, which are altered in chronic disease states [58]. The main metabolic reaction in the arginine metabolism is its conversion to nitric oxide (NO) and citrulline by NO synthase [59], which regulates NO bioavailability and, therein, its pleiotropic activities [60]. The presence of arginine among the most distinctive analytes in participants with F-T2DM suggests a role for arginine metabolism in muscle homeostasis and frailty [61]. This is corroborated by the effects of l-arginine supplementation on physical function in age-related conditions, including chronic lung disease [62], congestive heart failure [63], and conditions associated with accelerated biological aging, such as long COVID [64].

Aminoadipic acid is a lysine metabolite that is released into the circulation following proteolysis [65]. Circulating levels of aminoadipic acid have been associated with low muscle mass in old Taiwanese men [66]. In vitro and in vivo models suggest that aminoadipic acid may increase insulin secretion as a compensatory mechanism to maintain glucose homeostasis in early insulin resistance [67]. Noticeably, aminoadipic acid concentrations in the top quartile were associated with a fourfold increase in T2DM risk over 12 years of follow-up in middle-aged participants of the Framingham Heart Study [67].

Aspartic acid participates in several cellular processes, such as the urea cycle and malate-aspartate shuttle. The latter mediates the transport of nicotinamide adenine dinucleotide (NAD) reducing equivalents between the cytoplasm and the mitochondrial matrix, and modulates the NAD/NADH ratio, a critical regulator of pro-longevity sirtuin deacetylases [68,69]. Aspartic acid also plays a role in muscle energy metabolism [70,71] and counteracts lipopolysaccharide-induced muscle atrophy in a piglet model [72].

Tryptophan is an essential amino acid with both gluconeogenic and ketogenic properties that exerts multiple activities related to growth, mood, behavior, and immune function [73]. Tryptophan metabolism involves two main pathways, the tryptophan–kynurenine and tryptophan–methoxyindole pathways, that lead to the synthesis of NAD, serotonin, and melatonin [73]. A perturbed tryptophan metabolism has been described in several age-related conditions, including cardiovascular disease [74,75], T2DM [76], and depression [77]. Tryptophan and its associated metabolites have also been associated with low muscle mass [78,79], poor muscle quality [80], and frailty [81] in independent cohorts.

Cystine is a sulfur-containing amino acid derived from the oxidation of two cysteine molecules. Cystine transport into mammalian cells regulates cysteine supply and the bioavailability of essential molecules, such as taurine, glutathione, coenzyme A, and inorganic sulfur [82,83]. Altered plasma cystine levels were reported in aging and in conditions associated with increased oxidative stress [84,85]. In older adults with breast cancer, higher levels of cystine and 3-methylhistidine were associated with frailty [86]. Low plasma cystine was found in conditions characterized by progressive skeletal muscle catabolism, including cancer and HIV infection [87].

Taurine is the most abundant free amino acid in several tissues, including the skeletal muscle, which stores 70% of total body taurine [88]. In muscle, taurine participates in the regulation of ion transport, membrane stability, mitochondrial function, redox and osmotic balance, calcium handling, and muscle contractility [89,90,91]. Tissue taurine depletion accelerates skeletal muscle senescence and shortens lifespan in animal models [92]. For its multiple anti-aging activities, taurine has also been proposed as a possible remedy against sarcopenia [93]. 

Unexpectedly, branched-chain amino acids (BCAAs) were not included among the discriminating metabolites by the PLS–DA models. The only exception was isoleucine, which was selected among the variables that differentiated F-T2DM from PF&S (Figure 1). BCAAs and some of their metabolic products act as signaling molecules and metabolic rheostats, and regulate several biological processes ranging from protein synthesis to insulin secretion [94]. Circulating levels of BCAAs and their metabolites have been associated with muscle mass in older adults with functional impairment and low muscle quality [78,80]. Low non-fasting concentrations of leucine and isoleucine were detected in plasma from Norwegian community-dwelling older adults with sarcopenia [95]. Discrepancies with our findings may be due to differences in operational definitions of frailty and the experimental protocols adopted, as well as to heterogeneity in eating habits among participants of the different studies.

Collectively, our results suggest that alterations in arginine metabolism, redox balance, muscle metabolism, and turnover characterize the metabolic profile of older adults with frailty compared with controls. Participants with F-T2DM may have more pronounced muscle decay than PF&S and control participants, as highlighted by the co-occurrence of high 3-methylhistidine and low cystine levels. This may be due to the synergistic negative effects of frailty and diabetes on muscle metabolism [96]. A perturbed arginine metabolism characterizes both frailty phenotypes, with specific patterns in F-T2DM (higher arginine levels) and PF&S (higher citrulline) [21] (Figure S1). Further studies are needed to comprehensively assess arginine metabolism, including methylarginines and markers of NO bioavailability, due to the critical role played by this metabolic pathway on endothelial function and physical performance [60,97].

The present study has limitations that should be acknowledged. The cross-sectional design does not allow the temporal relationship between changes in amino profiles and frailty status to be established. A fairly large number of variables were evaluated in a relatively small sample. To cope with this issue, we adopted a PLS–DA approach, which is ideal for analyzing matrices containing highly correlated variables. BIOSPHERE and MetaboFrail enrolled older adults from three European countries. However, most participants were Caucasian; thus, findings may not be generalized to other ethnic groups. Dietary habits may influence circulating amino acid levels, but no nutritional data were collected. However, variations in blood amino acid concentrations do not necessarily reflect changes in amino acid intake [98]. Appendicular lean mass was available for BIOSPHERE participants and controls. Thus, it was not possible to ascertain whether markers of muscle turnover were associated with lean mass in participants with F-T2DM. Finally, although a quite large number of amino acids were evaluated, we cannot exclude that adding other mediators (e.g., amino acid derivatives such as arginine metabolites) might allow a more accurate classification of participants.

## 5. Conclusions

In the present study, we showed that specific amino acid profiles are associated with a distinct operational definition of frailty. Our findings also offer insights on the mechanisms potentially involved in the pathophysiology of frailty with or without T2DM, such as perturbations in muscle energy and interorgan metabolic pathways, alterations in arginine/NO metabolism, and oxidative stress. Further studies are needed to determine the contribution of individual pathways to the phenotypic manifestations of frailty, in order to develop targeted interventions.

## Figures and Tables

**Table 1:** Baseline Characteristics of Study Participants According to Frailty Categories.

Characteristic	PF&S (*n* = 94)	F-T2DM (*n* = 66)	Controls (*n* = 40)	*p*
Age, years	77.3 (4.9)	76.5 (4.5)	74.4 (3.9) *	0.0001
Women, *n* (%)	69 (73.4)	32 (48.5)	16 (40.0)	0.0001
BMI, kg/m^2^	30.0 (5.0)	29.2 (5.0)	26.6 (2.4) *	<0.0001
SPPB summary score	7.2 (1.2)	8.6 (2.9) ^§^	11.4 (0.8) *	<0.0001

Data are expressed as mean (standard deviation) unless otherwise specified. * *p* < 0.05 vs. PF&S and F-T2DM; ^§^ *p* < 0.05 vs. PF&S. Abbreviations: BMI, body mass index; F-T2DM, pre-frailty/frailty with type 2 diabetes mellitus; PF&S, physical frailty and sarcopenia; SPPB, short physical performance battery.

## Data Availability

Due to privacy or ethical restrictions, the data presented in this study are available from the corresponding author upon reasonable request pending approval by the MetaboFrail Scientific Committee.

## Supplementary Materials

The following supporting information can be downloaded at: https://www.mdpi.com/article/10.3390/metabo13040542/s1, Figure S1: Serum amino acid concentrations in study participants according to frailty category.
