# Supplementary material for: Amino Acid Profiles in Older Adults with Frailty: Secondary Analysis from MetaboFrail and BIOSPHERE Studies
Source: Metabolites. 2023 Apr 10;13(4):542. doi: 10.3390/metabo13040542 (PMC10147014; doi:10.3390/metabo13040542)
Supplement: Supplementary file 1 [file metabolites-13-00542-s001.zip › metabolites-2290305-supplementary.pdf]

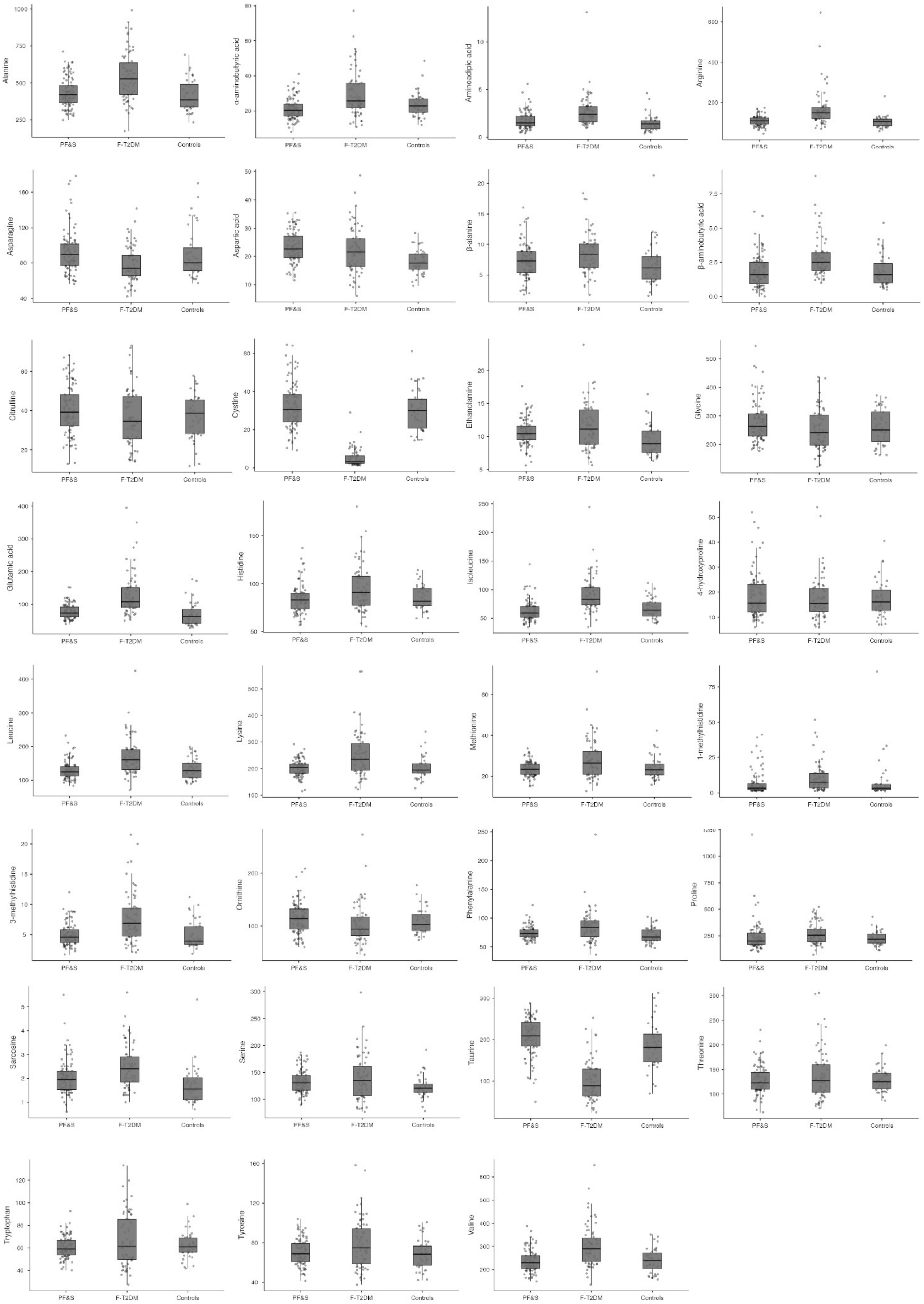

Figure S1. Serum amino acid concentrations in study participants according to frailty

category. Values are reported in μmol/L. Box plots show the median serum concentration (bold horizontal line), interquartile range (box), and total range of concentrations (bars).

Abbreviations: F-T2DM, pre-frailty/frailty with type 2 diabetes mellitus; PF&S, physical frailty and sarcopenia.
